# Role of Nrf2 in Pancreatic Cancer

**DOI:** 10.3390/antiox11010098

**Published:** 2021-12-30

**Authors:** Marta Cykowiak, Violetta Krajka-Kuźniak

**Affiliations:** Department of Pharmaceutical Biochemistry, Poznan University of Medical Sciences, 4, Święcickiego Street, 60-781 Poznan, Poland; marta.cykowiak@ump.edu.pl

**Keywords:** Nrf2, pancreatic cancer, oxidative stress, ROS, inhibitors and inducers of Nrf2

## Abstract

Pancreatic tumors are a serious health problem with a 7% mortality rate worldwide. Inflammatory processes and oxidative stress play important roles in the development of pancreatic diseases/cancer. To maintain homeostasis, a balance between free radicals and the antioxidant system is essential. Nuclear Factor Erythroid 2-Related Factor 2/*NFE2L2* (Nrf2) and its negative regulator Kelch-Like ECH-Associated Protein 1 (Keap1) provide substantial protection against damage induced by oxidative stress, and a growing body of evidence points to the canonical and noncanonical Nrf2 signaling pathway as a pharmacological target in the treatment of pancreatic diseases. In this review, we present updated evidence on the activation of the Nrf2 signaling pathway and its importance in pancreatic cancer. Our review covers potential modulators of canonical and noncanonical pathway modulation mechanisms that may have a positive effect on the therapeutic response. Finally, we describe some interesting recent discoveries of novel treatments related to the antioxidant system for pancreatic cancer, including natural or synthetic compounds with therapeutic properties.

## 1. Introduction

### 1.1. Pancreatic Cancer

**Figure 1:**
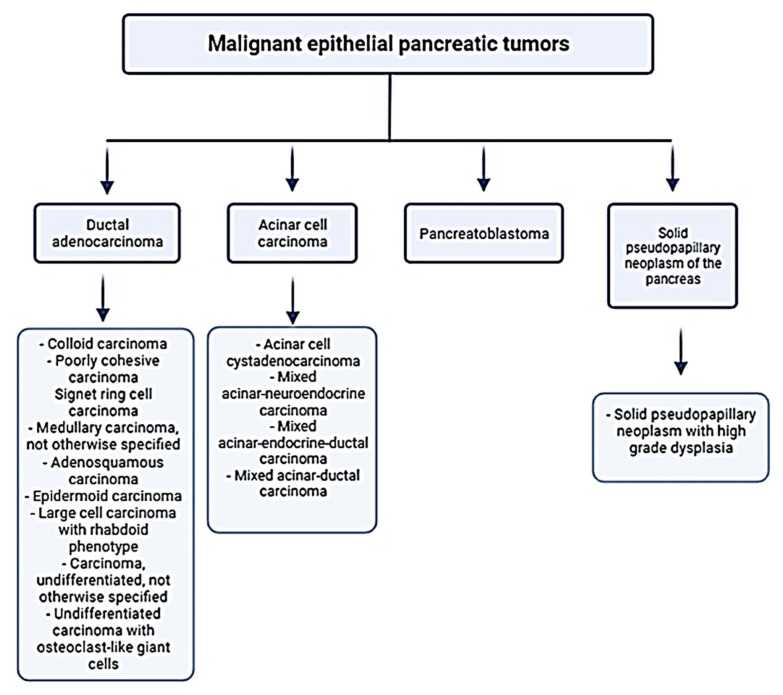
WHO classification of malignant epithelial pancreatic tumors [7].

Pancreatic cancer is one of the most malignant neoplasms in the world, with current statistics indicating that it ranks third after colon and lung cancer with a mortality rate of about 6% in Western Europe and the USA and about 7% worldwide. It is worth indicating that pancreatic cancer is prognosticated to take over the second position by 2030 [1]. The pancreatic cancer incidence rate is practically equal to that of mortality due to the absence of early symptoms as well as effective detection and treatment methods [2]. Furthermore, the evidence of risk factors for the development of pancreatic cancer is poor and does not adequately explain its development: currently, we can only identify risk factors in around 40% of cases [3]. The main environmental risk factors for pancreatic cancer, responsible for the development of approximately 30% of cancers, are: tobacco use (smoking habits are considered responsible for 20–35% of pancreatic cancer cases), alcohol consumption (alcohol consumption is responsible for 60–90% of chronic pancreatitis), chronic pancreatitis, age (80% of cases occur between the ages of 60 and 80), obesity, and diabetes mellitus. From unmodifiable (hereditary) risk factors, we should point out five syndromes: HBOC (Hereditary Breast and Ovarian Cancer syndrome), HNPCC (Hereditary Non-Polyposic Colorectal Cancer or Lynch syndrome), FAP (Familial Adenomatous Polyposis), PJS (Peutz–Jeghers Syndrome), FAMMM (Familial Atypical Multiple Mole Melanoma syndrome)—hereditary pancreatitis and cystic fibrosis [3]. In addition, research has confirmed that a diet too rich in animal fats and carbohydrates raises the risk of disease development [4]. The most common type of pancreatic cancer is pancreatic ductal adenocarcinoma (PDAC), and it accounts for approximately 85% of all pancreatic cancers [5]. We present the WHO classification of malignant epithelial pancreatic tumors based on the histological type of the tumor and various subclassifications in Figure 1. Moreover, the literature’s data indicate that approximately 95% of pancreatic malignant neoplasms are in the exocrine glands and originate mainly from ductal cells. About 90% of these tumors are diagnosed in the head of the pancreas. In 5% of pancreatic cancers, the cancer cells originate in the endocrine part of the pancreas and are called pancreatic neuroendocrine tumors (NETs) [6].

### 1.2. Oxidative Stress and Signal Transduction in Pancreatic Cancer

Oxidative stress usually results from an imbalance between the generation of reactive oxygen species (ROS) and the antioxidant defense systems of the cells. Several data indicate that oxidative stress plays a key role in the pathogenesis of pancreatitis, which in turn is an important risk factor for the development of pancreatic cancer [8,9]. In the inflammatory environment of pancreatitis, inappropriate activation of digestive enzymes within acinar cells, along with aberrant pancreatic enzyme secretion and increased inflammatory responses, can stimulate ductal metaplasia, which is considered the main origin of pancreatic preneoplastic lesions that develop into PDAC. However, the link between recurrent acute pancreatitis and pancreatic cancer still remains unclear. Several studies have shown that recurrent episodes of pancreatitis set into motion various inflammatory pathways which can lead to immunological and inflammatory responses. This in turn leads to increased stellate cell activation and fibrotic tissue formation, well-established hallmarks of pancreatic cancer [10]. Moreover, the oxidation of DNA and the subsequent mutation of genes, membrane disintegration, and oxidative stress, which causes protein misfolding, can promote carcinogenesis [8].

Signaling pathways coordinate communication between the cell surface and nucleus, between different cells, and between cells and the extracellular matrix. Aberrant signaling of just one pathway can have huge implications on wider signaling networks that consequently promote cancer progression and metastasis [11]. These two processes are dependent on angiogenesis, which includes proliferation, migration, and differentiation of endothelial cells. Furthermore, protein kinases and phosphatases, which coordinate the processes, play key roles, e.g., phosphorylation on serine, threonine, or tyrosine residues, which favors protein–protein interactions in signal transduction pathways. However, phosphorylation/dephosphorylation also promote signal activation by mechanisms independent of protein–protein interactions per se. In addition to protein phosphorylation, accumulating experimental evidence points to the involvement of redox-based post-translational modifications in angiogenesis, which may be associated with the formation of reactive oxygen species [12,13,14]. Several studies confirm that oxidative stress activates the Nrf2-ARE signaling pathway, which ultimately leads to the transcription of genes encoding antioxidant and detoxification proteins, such as Superoxide Dismutase (SOD) and Glutathione S-Transferase (GST), respectively. ROS-generating agents or agents that abrogate the inherent antioxidant systems could increase endogenous ROS, causing the level to exceed the cellular threshold, thereby inducing cell death. This notion is the so-called ROS threshold concept in cancer therapy [15,16,17,18,19].

Signal transduction involving oxidative stress and the Nrf2 pathway has emerged as a research area of intense interest in pancreatic cancer. Herein, we review the molecular details of Nrf2 structure/function and activation, describe recent findings that implicate the Nrf2 signaling in pancreatic cancer initiation/progression, and catalogue a variety of natural products and substances that impact the system and show therapeutic promise.

## 2. Molecular Aspects of Nrf2 Activation

The Nrf2 transcription factor is one of the central regulators of the oxidative stress response. Nrf2 plays an important role in maintaining homeostasis by inducing the expression of more than 250 target genes that control oxidation/reduction processes, metabolism of xenobiotics, DNA repair, and carbohydrate and lipid metabolism, in addition to ensuring proteostasis [20]. Furthermore, Nrf2 was found to be necessary in maintaining pancreatic cancer proliferation by regulating mRNA translation [21].

The transcriptional factor Nrf2 is a member of the Cap ‘n’ Collar (CNC) family with a basic leucine zipper region (bZip). There are also two other members of the Nrf subfamily: Nrf1 and Nrf3 [22]. The first one, Nrf1, functions as a transcription factor which activates the expression of some key metabolic genes regulating cellular growth and nuclear genes required for respiration, heme biosynthesis, and also mitochondrial DNA transcription and replication [23]. The second one, Nrf3, has functional significance in cancer; to be specific, high Nrf3 mRNA levels are induced in many cancer types, such as colorectal cancer and pancreatic adenocarcinoma, and are associated with poor prognosis [24].

**Figure 2:**
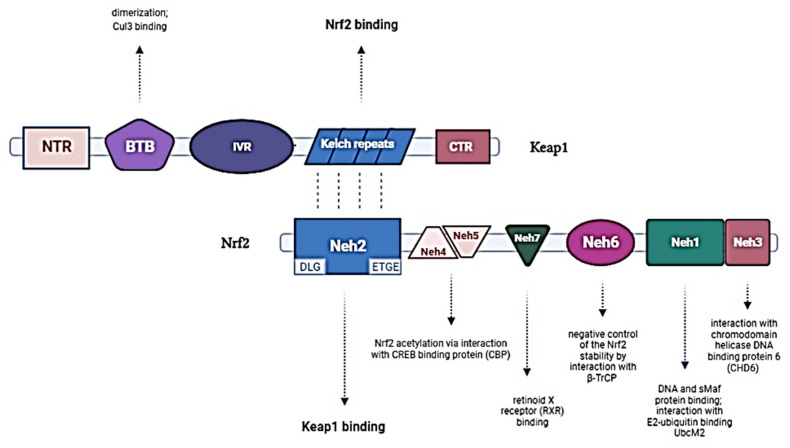
Domain structures of the Keap1 and Nrf2 proteins and their interactions. Keap1 possesses five domains: amino terminal region (NTR), a broad complex, tramtrack, bric-a-brac (BTB) domain, an intervening region (IVR), six Kelch domains, and the C-Terminal Region (CTR). The BTB domain homodimerizes with Keap1 and contributes to the interaction of IVR with Cul3/RBX1 complex. The Kelch domain and CTR mediate the interaction with Nrf2. Nrf2 has seven domains: Neh1–Neh7. Neh1 contains a basic leucine zipper (bZip) motif, where the basic region is responsible for DNA binding, and the Zip dimerizes with other binding partners such as sMAFs. Neh2 contains ETGE and DLG motifs, which are required for the interaction with Keap1. Neh3, 4, and 5 domains are transactivation domains of Nrf2. Neh4 and 5 domains interact with CREB binding protein (CBP). Neh6 contains two βTrCP degrons DSGIS and DSAPGS that are responsible for the β-TrCP-mediated proteasomal degradation. Neh 7 mediates interaction with retinoic X receptor alpha (RXRα), which represses Nrf2 activity.

The electrophoretic Nrf2 mobility indicates a 96–118 kDa molecular weight despite having 605 amino acid residues. It is caused by post-translational modifications such as phosphorylation [25] and the abundance of acidic residues in its structure [26]. The structure of the Nrf2 protein consists of seven conserved Nrf2-ECH homology (Neh) domains, Neh1–Neh7, presented in Figure 2, important in its activity and repression. The key domain is the Neh1 region which includes the CNC-bZip. This structure enables Nrf2 binding to the nuclear DNA, after its prior translocation to the cell nucleus. Neh2 and Neh6 are degron regions (an amino acid sequence or structure in a protein involved in its degradation), targeted by Keap1 and βTrCP. Neh4 and Neh5 domains allow binding with the CREB protein (cAMP response element-binding protein), as well as enhancing the transcriptional activity of Nrf2. Finally, Neh7 domain is implicated in Nrf2 repression by RXRα protein [27].

Some fundamental aspects of the Nrf2 structure and function have been previously established, such as binding to Kelch-like ECH-Associated Protein 1 (Keap1) that sequesters the molecule in the cytoplasm [28,29]. The structures of these two proteins, Nrf2 and Keap1, and their dependencies are also presented in Figure 2. In homeostatic conditions, Nrf2 resides in a molecular complex with a negative regulator-Keap1, which controls the expression level of Nrf2 by regulating its ubiquitination and degradation [30]. Thus, in the following sections the focus is on this interaction and its regulation.

### 2.1. Nrf2-Keap1 Interaction Interface and Its Regulation

The results of nuclear magnetic resonance spectroscopy have shown that the Neh2 region of Nrf2 is predicted to be intrinsically disordered but capable of binding to the full-length Keap1 protein at low nanomolar concentrations [31]. This binding was replicated by two motifs, a 16-residue peptide incorporating amino acids 69–84 of Nrf2, which flank the conserved ETGE motif and DLG motif [31]. The conserved DLG motif in the Neh2 region of Nrf2 was identified as a second independent binding site with 100-fold weaker affinity for Keap1 [32,33].

The two separate DLG and ETGE motifs allow binding of a single Nrf2 molecule to the two Kelch domains present in the Keap1 dimer. The mechanism called hinge and latch has been suggested to explain the interactions between these two proteins. In this mechanism, the high-affinity ETGE motif acts as a hinge anchored to the first Kelch domain, whereas the weaker DLG motif is engaged as the latch to maintain minimal housekeeping expression levels of the Nrf2 protein [32].

Under nonstress conditions, Keap1 is associated with the Keap1-CUL3-RBX1 E3 ubiquitin ligase complex, where Keap1 functions as the substrate adaptor, RBX1 binds to the ubiquitin loaded E2-ubiquitin conjugating enzyme, and CUL3 provides the scaffold which joins Keap1 and RBX1. This complex functions to correctly orientate the Nrf2-bound Keap1 and the E2-bound RBX1 to facilitate the ubiquitination of Nrf2 [33].

More recently, the importance of Nrf2 in cancer has been established. Somatic mutations in the DLG and ETGE motifs of Nrf2, with very high frequency, occur in numerous cancer cells, leading to the disruption of the two-site binding of Keap1–Nrf2 and constitutive accumulation of Nrf2 [34]. This constitutive activation of Nrf2 in cancers induces prosurvival genes, such as heme oxygenase (*HO-1*), *GSTP*, *GSTA1*, NAD(P)H:quinone oxidoreductase 1 (*NQO1*), and promotes cancer cell proliferation by metabolic reprogramming, repression of cancer cell apoptosis, and enhancement of the self-renewal capacity of cancer stem cells [35].

### 2.2. Canonical and Noncanonical Nrf2 Activation

**Figure 3:**
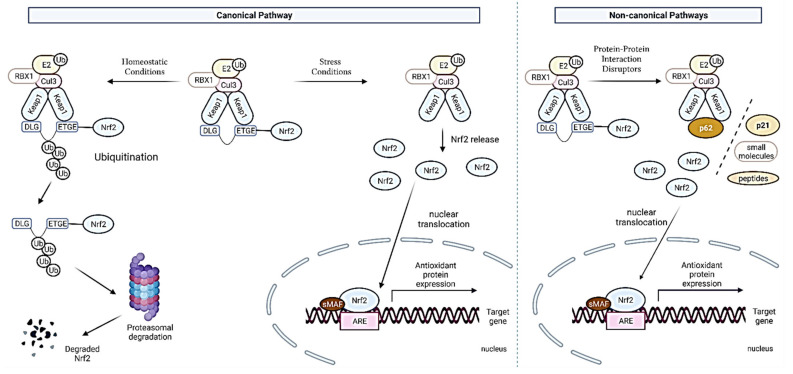
Canonical and noncanonical pathways of Nrf2 activation.

So far, two mechanisms of interaction between Nrf2 and Keap1 have been described and they are presented in Figure 3. The mechanism of their activation is called the canonical pathway of Nrf2 activation. The first, dependent on the Keap1, the so-called “hinge and latch” model, assumes that Keap1 interaction with the ETGE domain acts as a hinge and weaker cooperation with the DLG motif acts as a latch [36], as previously described. Under stress conditions, electrophilic compounds modify thiol residues, and the DLG motif dissociates from Keap1, Nrf2 is released from the Keap1-Cul3-RBX1 complex and translocates to the nucleus where it binds to an antioxidant response element (ARE), activating the genes transcription [37,38].

A model independent of Keap1, suggests that the Neh6 domain binds to the DSGIS and DSAPGS beta-TrCP motifs (beta-transducin repeat protein), which in turn is a substrate receptor for the Skp1-Cul1-Rbx1/Roc1 ubiquitin ligase complex, which directs the ubiquitination of Nrf2 [39]. Nrf2 phosphorylation in the Neh6 domain by glycogen synthase kinase-3 regulates the recognition of the Neh6 domain by beta-TrCP [37,40].

In the nucleus, Nrf2 combines with small Maf proteins (MafF, MafG, and MafK) through its bZip domains and binds to the ARE sequences, inducing its transcriptional activation [41]. Moreover, the acetylation by p300/CBP and SUMOylation by UBC9 are needed for Nrf2 binding to the ARE sequence [42].

The noncanonical Nrf2 activation pathway affects the complex of Nrf2 with the Keap1 inhibitor protein through the interaction of Nrf2 or Keap1 with other proteins such as p62, DPP3, WTX, prothymosin α, PALB2, and p21. Through these interactions with proteins, Nrf2 is protected against ubiquitination and the degradation of the 26S proteasomes. This fact ensures the translocation of Nrf2 to the nucleus and its activation. The most studied mechanism by the noncanonical pathway is the activation of Nrf2 by the p62 protein (SQSTM1). p62 is a classical receptor of the autophagy process, which is responsible for the removal of damaged proteins and organelles, including oxidatively damaged proteins and dysfunctional mitochondria. p62 is a multidomain protein that can interact with a protein targets, including Keap1. Similar to the interaction of Keap1 with Nrf2, the p62 protein is capable of interacting with Keap1 through the KIR domain of p62. In addition, phospho-p62 competes with the ETGE motif of Nrf2 for Keap1 binding. The interaction of Keap1 with p62 induces autophagy-dependent Keap1 degradation and the subsequent stabilization and activation of Nrf2 [42,43,44,45].

The Nrf2 activation dependent on p62 increases the Nrf2 activation and expression of NQO1, GST, and antiapoptotic proteins such as Bcl-2 and Bcl-xL, decreasing ROS levels and protecting the cell against oxidative stress [46,47]. However, sustained Nrf2 activation by impairment in autophagy and increase in p62 phosphorylation promotes antineoplastic drug chemoresistance as well as cancer cell proliferation, whereas mutation in KIR domain in p62, which prevents Keap1-p62 interaction, is associated with an ROS increase [42,44,48,49,50,51].

Recent research has highlighted DDP3, an aminopeptidase that is involved in the regulation of oxidative stress through modulation of the Nrf2 pathway by direct interaction with Keap1 via its ETGE motif and the Kelch domain of Keap1. In addition, overexpression of DDP3 reduces the ubiquitination of Nrf2 and enhances the translocation and nuclear activation of Nrf2, which protects cells from oxidative stress and prevents cell death by reducing ROS [52,53].

In vitro and in vivo data demonstrated that prothymosin α is a nuclear protein that binds to the Kelch domain in Keap1 and enhances Nrf2 activation and HO-1 expression [54,55]. The p21 protein also regulates Nrf2 activation by blocking the DLG motif binding site in Keap1. Additionally, p21 overexpression increases Nrf2 activation and expression of both HO-1 and NQO1, and this in turn may guarantee the reduction of ROS levels and protect cells from oxidative stress [42,56].

## 3. Dual Role of Nrf2 in Pancreatic Cancers

As mentioned previously, redox status imbalance is a common feature of various cancers types. Permanently high ROS levels in tumor cells are observed due to the oncogene activation, hypoxia, increased metabolic rates, anchorage-independent growth, and mitochondrial and/or peroxisomal dysfunction [57]. In these circumstances, Nrf2 plays a key role in the antioxidant response. Its function as a major regulator of redox processes can be either beneficial or prejudicial depending on the stage of tumorigenesis in pancreatic cancer cells [58]. In the early phase of pancreatic carcinogenesis, Nrf2 activation prevents carcinogen-induced carcinogenesis by activating the transcription of genes that regulate detoxification, antioxidation, and immune surveillance [59]. However, at the stage of cancer progression, the activating mutations in Nrf2 and loss-of-function mutations in Keap1 lead to the disruption of Keap1-Nrf2 binding and constitutive activation of Nrf2, increasing the expression of genes necessary for cancer cell proliferation, ferroptosis, angiogenesis, senescence, autophagy, angiogenesis, drug resistance, and metastasis [59]. In PDAC, gene mutations in the Keap1/Nrf2 pathway are rare, but Nrf2 expression levels are high in over 93% of pancreatic adenocarcinomas [60]. The Nrf2 overexpression is believed to be a consequence of the near-universal presence of oncogenic KRAS gene mutations and downstream activation of the MAPK pathway and high levels of c-Myc [61,62]. What is worth noting, the high nuclear expression of Nrf2 correlates with reduced survival rates of pancreatic cancer patients. Recently, it has also been found that elevated Nrf2 levels in early precursor lesions in the pancreas contribute to pancreatic carcinogenesis [63,64]. Thus, in the next section, we give a comprehensive overview of the dual roles of the Keap1-Nrf2 pathway in pancreatic cancer.

### 3.1. Nrf2 as Tumor Suppressor in Pancreatic Cancer

During the early stage of pancreatic carcinogenesis, Nrf2 exerts a tumor-suppressive role by binding to antioxidant response elements and activating its downstream target genes (such as *NQO1*, *SOD1*, *HO-1*, *ATF3*, *IL-17D*, and *SQSTM1/p62*) that regulate the cellular antioxidant/detoxification response, immune surveillance, and autophagy [65,66,67,68]. Evidence showing the preventive effects of Nrf2 on pancreatic carcinogenesis and pancreatic tumor growth and metastasis has previously been provided. Kim et al. showed that the activation of Nrf2, caused by oxidative stress, protected pancreatic beta cells from damage and apoptosis, preventing pancreatic carcinogenesis induced by oxidants and carcinogens [69]. Furthermore, the Nrf2 activation by various natural products has also been found to inhibit pancreatic cancer growth and induce apoptosis, although the exact association of the activation of Nrf2 and the anticancer efficacy need to be further investigated [70,71]. Probst et al., found that RTA 405, an antioxidant inflammation modulator and Nrf2 activator, suppresses cancer cell survival and promotes apoptosis via downregulating the NF-κB activity [72]. In the next section, we present the exact results of studies using Nrf2 activators.

### 3.2. The Carcinogenic Role of Keap1-Nrf2 Pathway in Pancreatic Cancer

In contrast to the protective impact of Nrf2 on oxidative stress-induced carcinogenesis, a great number of studies have demonstrated a supporting role of the Keap1-Nrf2 pathway in pancreatic cancer development. It is widely accepted that Kras-mediated Nrf2 expression and activation leads to low intracellular ROS levels and promotes pancreatic tumorigenesis and metastasis, whereas Nrf2 inhibition blocks Kras-induced cell proliferation, tumorigenesis, and metastasis. Hamada et al. examined the role of Nrf2 in pancreatic carcinogenesis in a mouse model carrying pancreas-specific Kras and p53 mutations (KPC mouse model) [63]. They demonstrated that Nrf2 deletion in the KPC mice correlated with a decrease in the formation of precancerous lesions and reduced the development of invasive pancreatic cancer. Furthermore, two cell lines lacking the expression of Nrf2 (KPCN) and its downstream target genes, including *NQO1*, have been established. KPCN-derived cell lines revealed increased sensitivity to oxidative stress and chemotherapeutic agent. Interestingly, the simultaneous activation of Kras and Nrf2 by Kras mutation and Keap1 deletion, respectively, did not promote pancreatic cancer development but led to pancreatic atrophy [73].

Kha et al., demonstrated that the activation of Nrf2 protected premalignant pancreatic ductal epithelial (PDE) cells from apoptosis and accelerated the formation and growth of pancreatic tumors via induction of the expression of a splicing variant of ATF3 (activating transcription factor 3), termed ΔZip2 [74]. Furthermore, Nrf2 can also attenuate transforming growth factor-β1 (TGF-β1)-mediated growth inhibition of PDE cells by diminishing the expression levels of p21, phospho-p38, and 7hosphor-Smad3 [64]. Pancreatic stellate cells (PSC), a prominent stromal cell, contribute to the progression of PDAC. Wu et al., aimed to investigate the mechanisms by which PSC promote cell proliferation in PDAC cell lines, BxPC-3, and AsPC-1. They found that stromal-derived factor-1 alpha (SDF-1α) and interleukin-6 (IL-6) activated the Nrf2 pathway and induced PDAC cell proliferation via Nrf2-activated metabolic reprogramming and ROS detoxification [75]. Their follow-up study has demonstrated that the Stat3/Nrf2 pathway mediates Epithelial–Mesenchymal Transition (EMT, a key process for the metastatic cascade) induced by IL-6. Nrf2 has also been reported to induce EMT by regulating the cancer cells and macrophages interaction [76]. The increased intracellular ROS level caused by pancreatic cancer cells via lactate secretion activates Nrf2 induced macrophage M2 phenotype transformation and escalates vascular endothelial growth factor (VEGF) expression [77]. The cancer cell-educated macrophages then induce Nrf2 activation in pancreatic cancer cells via VEGF secretion and promote EMT. Not only is p62 an autophagy substrate that is used as a reporter of autophagy activity, but it was also shown to deliver ubiquitinated proteins, such as tau, to the proteasome for degradation [78]. Furthermore, p62 accumulation was demonstrated in PDAC and corresponded with increased expression of Nrf2 target genes, which are activated by p62, and *MDM2* [79]. Todoric et al., demonstrated that pancreatitis-induced accumulation of the autophagy substrate p62/SQSTM1 in the context of oncogenic KRAS promotes progression to pancreatic ductal adenocarcinoma. This p62 function relies on Nrf2-driven induction of *MDM2* and both *p53*-dependent and -independent activity of *MDM2* [79]. In a very recent study, Su et al. showed that autophagy inhibition upregulates micropinocytosis (MP), which provides nutrients supporting the growth of autophagy-deficient cancers [80]. The autophagy to MP switch depends on the Nrf2-driven induction of MP-related proteins. This switch may be evolutionarily conserved and not only cancer related and probably is a consequence of the activation of Nrf2 by the autophagy adaptor p62/SQSTM1. Nrf2 activation by oncogenic mutations, hypoxia, and oxidative stress also results in MP upregulation. The inhibition of MP in autophagy-compromised PDAC elicits dramatic metabolic decline and regression of transplanted and autochthonous tumors, suggesting a promising therapeutic use by combining autophagy and MP inhibitors in the clinic [80].

An important aspect of the activation of Nrf2 in pancreatic cancer is chemoresistance [81]. The Keap1-Nrf2 pathway is involved in pancreatic cancer chemoresistance by regulating the expression of drug resistance-associated genes (*MRP1*, *MRP2*, *MRP3*, *MRP4*, *MRP5*, and *ABCG2*) [68,82,83] and previously mentioned cytoprotective antioxidant genes. Moreover, Duong et al. observed that upregulation of aldehyde dehydrogenase 1 family, member A1 (ALDH1A1) and aldehyde dehydrogenase 3 family, member A1 (ALDH3A1) caused by Nrf2 may also contribute to drug resistance in pancreatic cancers [84]. Nevertheless, chemotherapies themselves, e.g., gemcitabine have been found to increase Nrf2 expression. This effect was inhibited by pretreatment with Nrf2 inhibitors, thus enhancing the sensitivity of pancreatic cancer cells to chemotherapy [85]. With reference to the abovementioned mechanisms, in the next section we present the selected Nrf2 inhibitors in pancreatic cancer therapies.

## 4. Therapeutic Strategies Targeting Nrf2 in Pancreatic Cancers

Natural compounds derived from medicinal plants have been known to possess therapeutic properties for many years and have been employed to successfully treat a great variety of human disorders. Recently, many plant extracts and discrete phytochemicals have emerged as promising chemopreventive and anticancer agents. Currently, many of them are under clinical trial investigation or already being administered in established therapeutic regimens. Extensive research has been pursued with a view to finding natural compounds as well as synthetic compounds with modulatory properties on Nrf2, known to be often overexpressed in many types of cancers, including pancreatic cancers. In the next section, we focus on some of the most recent and significant discoveries in pancreatic cancer therapies targeting Nrf2. We summarized them in the Table 1. 

### 4.1. Natural Compounds

#### 4.1.1. Natural Compounds with Inhibitory Effects on Nrf2

##### Trigonelline

Trigonelline (TRG) is an alkaloid abundantly present in many plants such as hemp seed, coffee beans, oats, and garden peas, firstly isolated from *Trigonella foenum-graecum* [85]. Arlt and coworkers confirmed that in three PDAC cell lines (Panc1, MiaPaCa-2, and Colo357) high basal Nrf2 activity correlated with the protection from TRAIL- and Etoposide-induced apoptosis by increasing the expression of proteasomal genes [86]. This study showed that submicromolar doses of TRG efficiently inhibited Nrf2 nuclear accumulation and proteasome activity, suppressing their protective functions in vitro and in vivo. Thus, the use of TRG might be considered as adjuvant treatment for patients with limited therapeutic options.

##### Brusatol

The quassinoid brusatol (BR) was first isolated and characterized from the seeds of *Brucea sumatrana* in 1968 [87]. Firstly, it was used as a treatment for amebiasis: BR was quickly recognized as a potent anticancer agent, but the molecular target of this compound has long been elusive [87]. Ren and coworkers in 2011 described for the first time how Nrf2-mediated mechanism of defense is suppressed by BR [88]. An in vitro study on pancreatic cell lines PATU-8988 and Panc1 further indicated that BR monotherapy resulted in substantial cytotoxicity in these cells [89]. Furthermore, the follow-up investigation showed an enhanced therapeutic effect of gemcitabine in combinatorial treatment with BR, evidenced by increased apoptosis and diminished xenograft formation [90]. Xiang et al. showed that BR inhibits growth and induces apoptosis in pancreatic cancer cells-PATU-8988 and Panc1, through the activation of the JNK (c-Jun N-terminal Kinase)/p38 MAPK (mitogen-activated protein kinase) and subsequent inhibition of NF-κB/STAT3/BCL2 signaling [89]. These observations were further confirmed in 2018 by the same group as BR reduced the Nrf2 protein content in a Keap1-independent manner and decreased the expression of genes related to the multiple drug resistance (MDR) family involved in gemcitabine resistance of pancreatic cancers [90].

##### Digoxin

Cardiac glycosides are a class of glycosides with strong cardiac functions, mainly used in the treatment of chronic cardiac insufficiency and heart failure. Among them, digoxin (DGX) is mainly used to treat heart failure. Interestingly, several studies have reported that digoxin exerted antitumor activities by inhibition of proliferation and induction of apoptosis, therefore supporting its potential use for cancer therapy [91]. In a recent study, Zhou et al. showed that DGX increased the sensitivity of SW1990/Gem (Gemcitabine resistant) cells to gemcitabine by 5.27-fold, 13.97-fold, and 35.83-fold at the doses of 20, 40, and 80 nM, while it increased the sensitivity of Panc-1/Gem (Gemcitabine resistant) cells to gemcitabine by 3.91-fold, 7.17-fold, and 20.43- fold at the doses of 20, 40, and 80 nM, respectively [92]. Furthermore, the same study demonstrated that gemcitabine in combination with DGX dramatically increased the number of cells undergoing apoptosis and inhibited cell colony formation when compared with gemcitabine single treatment. These results were correlated with the marked decrease in total and nuclear protein levels of Nrf2 in SW1990/Gem and Panc-1/Gem cells. Interestingly, digoxin at tested doses could not significantly inhibit total and nuclear Nrf2 protein levels in SW1990 and Panc-1 cells.

##### Ailanthone

Ailanthone (Aila) is a natural compound extracted from the tree *Ailanthus altissima*. This phytochemical, in traditional Chinese medicine, has been employed to treat several disorders including cancer [93]. In recent years, it has been demonstrated that Aila was able to inhibit Nrf2 and Yes-Associated Protein 1(YAP) expression in diverse cell models and suppress the proliferation of different cancer cell lines. This inhibition was usually accompanied by cell cycle arrest and apoptosis [94]. Grattarola et al., demonstrated that the Aila treatment led to the reduction of Nrf2 and YAP protein expression, as well as of the respective targets, GSTA4 and survivin in Panc1 cells [95]. The results confirmed that the reduction of Nrf2 and YAP expressions was accompanied by a reduction of the proliferative potential in intrinsically chemoresistant Panc1 cells. The group suggested that in PDAC cells the regulation of Nrf2 and YAP protein expression did not depend on the mRNA synthesis but rather on post-translational modifications. Following this observation, treatment with MG132, a proteasome inhibitor, increased Nrf2 and YAP expression levels in all PDAC cell lines analyzed in this study.

#### 4.1.2. Natural Compounds with Activation Effects on Nrf2

##### Curcumin

Curcumin (CUR), a natural pigment extracted from turmeric, is the primary active ingredient of this plant. The compound possesses a variety of pharmacological effects, such as antioxidative, anti-inflammatory, antitumor, antibacterial, free radical scavenging, and neuroprotective effects. Notably, CUR has proven to be an effective Nrf2 activator that intervenes in the interaction of Nrf2-Keap1 and has a positive effect on sestrin2 in the AKT-Nrf2 pathway. Recently, Fu and coworkers demonstrated that CUR enhances the antitumor growth effect of sestrin2 through the Nrf-2-Keap1/HO-1/NQO-1 signaling pathway in pancreatic cancer cells [96]. Previously, Pastorelli et al. observed, in clinical trials, that activating Nrf2, CUR downregulates NF-κB controlled genes involved in inflammation, proliferation, survival, invasion, angiogenesis, and metastasis [97]. Whereas inflammation within the pancreatic cancer microenvironment, seemingly due to NF-κB activation, has been linked to tumor progression and chemoresistance, these findings reinforce preclinical evidence that the complementary therapy to other chemotherapeutic agents with CUR exhibits beneficial efficacy and safety during anticancer therapy.

##### Sulforaphane

Sulforaphane (SFN) is a sulfur-containing isothiocyanate found in cruciferous vegetables (*Brassicaceae*). Recent studies have demonstrated that SFN potentially has numerous essential roles as an antimicrobial, antioxidant, anti-inflammatory and antioncogenic agent and as an epigenetic modulator [98]. SFN stabilizes and is an Nrf2 agonist and, as such, can indirectly influence the transcription of a battery of antioxidant enzymes [27]. Chen et al. demonstrated that SFN inhibited the growth of two pancreatic cancer cell-Panc1 and MiaPaCa-2 in a time- and dose-dependent manner [99]. Moreover, after SFN treatment, excessively generated reactive oxygen species activated adenosine 5′-monophosphate-activated protein kinase (AMPK) and subsequently increased the Nrf2 nuclear translocation, which suppressed pancreatic cancer cell proliferation. In a pancreatic cancer transgenic mouse model, SFN treatment (50 mg/kg, i.p.) inhibited tumor growth, consistent with the antiproliferative effects of SFN through ROS-activated AMPK signaling pathway and NRF2 nuclear translocation. In summary, they observed that the activation of Nrf2 by sulforaphane inhibited high glucose-induced progression of pancreatic cancer via AMPK-dependent signaling, by the inhibition of clone formation, the migration of pancreatic cancer, and the promotion of apoptosis.

##### Esculetin

Esculetin is found in various medicinal plants such as *Cichorium intybus* (Asteracea), *Artemisia capillaries* (Compositae), *Ceratostigma willmottianum* (Plumbaginaceae), *Citrus limonia* (Rutaceae). Arora et al., discovered a dihydroxy coumarin derivative, termed esculetin that directly binds to Keap1 and inhibits its binding to Nrf2 [100]. The Nrf2 released by esculetin reduced ROS level, inhibited cell growth, arrested cells at G1 phase, and induced apoptosis and the loss of mitochondrial membrane potential in Panc1 and MiaPaCa-2 cells. The authors have suggested that esculetin binds to Keap1 and inhibits its interaction with Nrf2 in pancreatic cancer cells. This thereby promotes nuclear accumulation of Nrf2 and induces antiproliferative and apoptotic response possibly by attenuating NF-κB. However, the selectivity of esculetin to Keap1 and its in vivo efficacy still need further investigation in clinical models.

##### Xanthohumol

Xanthohumol (XN), a prenylated chalcone, is the principal flavonoid found in the hop plant (*Humulus lupulus* L.). Therefore, it is an important ingredient in beer. Several studies evaluating the anticancer potential of XN showed its effectiveness in different cancer models in vitro and in vivo, partly via induction of the Nrf2 pathway [101]. XN was found to elevate the expression of Nrf2 and its target genes in MiaPaCa-1 and Panc1 cells [70,71]. This activation of the Nrf2 pathway affected the cell cycle distribution changes. Furthermore, its combination with phenethyl isothiocyanate inhibited proliferation in MiaPaCa-2 and Panc1 cells and increased Caspase-3 and LC3 proteins expression in Panc1 cell line.

##### Resveratrol

Resveratrol (RSV), a polyphenol compound found in certain plants and in red wine that has antioxidant properties and has been investigated for possible anticarcinogenic effects. Cheng et al., found that the Nrf2 pathway and the iron-sulfur protein Nutrient deprivation-Autophagy Factor-1 (NAF-1) negatively interact with one another upon ROS stimulation, which has crucial importance in promoting pancreatic cancer cell death. In this study, the elevated level of Nrf2 after treatment with RSV in Panc1 and MiaPaCa-2 was observed [102]. In two studies in which RSV was used in combination with other phytochemicals, a similar effect was confirmed. These results suggested that RSV promotes the apoptosis of pancreatic cancer cells via activation of the Nrf2 pathway and consequently downregulates the NF-κB activity [70,71].

##### Phenethyl Isothiocyanate

Phenethyl isothicyanate (PEITC) is a naturally occurring compound found in cruciferous vegetables. It is known as a dual activator of transcription factors Nrf2 and HSF1. In 2015, Ju et al., postulated that PEITC conjugates with GSH, which expression is regulated by Nrf2, to export it from cells, and circumvents cellular resistance to gemcitabine. The incubation of PDAC cells with 5 μmol/L PEITC for 3 h significantly depleted cellular GSH (80%), whereas incubation with 5 μmol/L PEITC for 24 h significantly increases the cellular ROS level. Furthermore, treatment with 5 μmol/L PEITC for 48 h resulted in an apoptosis level of approximately 60% in Panc-28, MiaPaCa-2, and AsPc-1 cell lines [103].

In a study from 2020, PEITC alone and in a combination with XN increased the level of nuclear Nrf2 and its binding to ARE sequence, increasing the expression of Nrf2-dependent genes. This activation by the combination of phytochemicals resulted in cell cycle distribution changes, correlated with increased Caspase-3 and LC3 proteins expression [70].

### 4.2. Synthetic Compounds

#### 4.2.1. Nrf2 Inhibitors

##### Dexamethasone

Dexamethasone is a corticosteroid (cortisone-like medicine or steroid) working on the immune system to help relieve swelling, redness, itching, and allergic reactions. Suzuki et al., in a very recent study reported that dexamethasone increases the effects of gemcitabine on pancreatic cancer stem cells. The group demonstrated that dexamethasone treatment reduced the expression of Nrf2, a key regulator of antioxidant responses, which was accompanied by a significant decrease in GSH in Panc1 cancer stem-like cells (CSLC) and PSN-1 CSLC [104]. Furthermore, dexamethasone increased the growth-inhibitory effects of gemcitabine and 5-fluorouracil, whereas N-acetyl-cysteine, an ROS scavenger, abolished this effect. Although dexamethasone alone did not increase ROS levels, dexamethasone promoted the increase in ROS levels, in an Nrf2-dependent manner, induced by gemcitabine and 5-fluorouracil. Moreover, the knockdown of the glucocorticoid receptor weakened the effects of dexamethasone on Nrf2 suppression and resulted in the loss of dexamethasone-induced chemosensitivity. This suggested that dexamethasone promotes chemotherapy-induced ROS production by suppressing the expression of Nrf2, most likely via the glucocorticoid receptor [104].

##### Dimethyl Fumarate

Dimethyl fumarate (DMF) is the methyl ester of fumaric acid and is named after the earth smoke plant (*Fumaria officinalis*). DMF is an oral drug that has been approved for the treatment of multiple sclerosis and psoriasis and has antioxidant properties [105]. Studies have shown that DMF exerts its protective effects by activating the Nrf2 antioxidant pathway and its anti-inflammatory effects by inhibiting NF-κB activity and reducing the expression of proinflammatory mediators [106]. In 2018, a group from France decided to ask whether 100 μM DMF would modulate the Nrf2/DJ-1 axis in nontumorigenic cells in a similar fashion to cancer cells. Treatment of nontumorigenic cells with increasing concentrations of DMF led to an increase in the protein expression levels of nuclear Nrf2 level and its downstream target, HO-1. In parallel, in pancreatic cancer cells Mia PaCa-2, a decrease in total Nrf2 and HO-1 was observed [107]. To understand the differential effect of DMF on Nrf2 activation in cancer and nontumorigenic cells, the expression of Keap1 and DJ-1 proteins, two partners of NRF2 targeted by DMF, was assessed. A clear decrease in DJ-1 protein levels was seen in cancer cells that were treated with DMF compared to those that were treated with DMSO, but not in normal cell lines. The results confirmed that DMF modulates Nrf2 and DJ-1 protein expressions in nontumorigenic cells differently than in cancer cells. 

#### 4.2.2. Compounds Interfering with Oncogenic Functional Interactors of the Nrf2/Keap1 Pathway

To date, it has been well established that many upstream regulators and downstream effectors can influence the biological effects exerted by and the activation status of the Nrf2/Keap1 signaling pathway. The list of these functional interactors is undoubtedly expanding; thus, in this section, we describe some of the most relevant oncogenic signaling pathways that impact on Nrf2 activation. It should be emphasized that the pharmacologic inhibition of these molecular targets has been largely exploited for drug repurposing, providing encouraging results in the context of therapy-resistant tumors.

##### Inhibitor of PI3K/DNA-PK–PIK-75

PIK-75 hydrochloride is a reversible DNA-PK and p110α-selective inhibitor, which inhibits DNA-PK, p110α, and p110γ. In 2014, Duong et al. demonstrated that PIK-75 decreased the Nrf2 protein levels and its transcriptional activity by proteasome-mediated degradation in human pancreatic cancer cell lines and a xenograft model [82]. Since gemcitabine resistance in pancreatic cancer is a significant problem, in part due to the upregulation of the Nrf2 activity, the PIK-75 was used as an adjuvant to gemcitabine. PIK-75 was able to counteract the increase in Nrf2 induced by gemcitabine and to significantly potentiate its antitumor effects both in vitro and in vivo. Notably, this study provided a strong mechanistic rationale to employ Nrf2-targeting agents in combination with gemcitabine for improving the clinical outcome of patients affected by otherwise resistant PDAC.

##### Inhibitor of Asparagine Synthesis Pathway-NSC84167

In a recently published study, Dai et al. identified the compound NSC84167 (7-oxo-7H-benzo[e]perimidine-4-carboxylic acid), selectively targeting Nrf2-activated pancreatic cancers by inhibiting the asparagine synthesis pathway [60]. The exposure of cells to NSC84167 for 48 h induced dramatic apoptosis in sensitive lines (high Nrf2 expression) but had no evident effect in the resistant line. Western blotting confirmed a dose-dependent effect in the sensitive lines, Panc1 and ASPC1, as measured by cleavages of PARP, caspase-9, and caspases-3. Results from previous studies have indicated that apoptosis observed in targeting the Nrf2 pathway is usually a consequence of ROS production; however, NSC84167 induced limited ROS in both sensitive and resistant pancreatic cancer cell lines. In addition, NSC48167 did not inhibit the transcriptional activity of Nrf2 or downstream NQO1 expression in either sensitive or resistant cell lines. The authors suggested that the effect appeared to be independent of Nrf2 transcriptional activity or ROS production. Previous studies have shown that activation of the Nrf2 pathway can reprogram cancer cell metabolism and upregulate multiple genes involved in the synthesis of serine, glycine, and asparagine, fueling cancer cell growth and survival [108,109,110]. Thus, they explored the inhibition of those processes as a possible mechanism of action of NSC84167. They found that NSC84167 inhibited asparagine de novo synthesis pathway by targeting protein translation and suggested that, mechanistically, NSC84167 could be activated by NQOl, which has been demonstrated to be a bioactivator of beta-lapachone.

**Table 1:** Therapeutic strategies targeting Nrf2 in pancreatic cancers.

**Natural Compounds**				
**Compound**	**Dosage**	**Model**	**Mechanism of Modulation**	**Reference**
**Trigonelline**	0.01–10 μM	Panc1, MiaPaCa-2, and Colo357 cell lines	a dose-dependent inhibition of ARE-driven luciferase expression; a decreased accumulation of Nrf2 protein in the nucleus; reduction of proteasome activity	[86]
**Brusatol**	0.5 μM	PATU-8988, BxPC-3 and Panc1 cell lines	inhibition of the Nrf2 pathway and increased ROS accumulation; abrogation of Gemcitabine-induced Nrf2 activation; decrease in mRNA and protein levels of Nrf2 target genes	[89]
	2 mg/kg i.p. once/day	Panc-1 xenografts	augmented antitumor activity of Gemcitabine	[90]
**Digoxin**	20 and 40 and 80 nM	SW1990 and Panc1 cell lines with induced gemcitabine-resistance	decrease in total and nuclear protein levels of Nrf2; an increase in the sensitivity to gemcitabine; an increase in the number of cells undergoing apoptosis and inhibition of cell colony formation compared with gemcitabine single treatment	[92]
**Ailanthone**	0.1–2 μg/mL	Panc1 cell line	post-translational downregulation of Nrf2 and YAP proteins, by targeting deubiquitinases	[95]
**Curcumin**		Panc1 and CFPAC-1 cell lines	enhanced antitumor effect of Sestrin2 through the Nrf-2-Keap1/HO-1/NQO-1 signaling pathway	[96]
	Meriva^®^, a patented preparation of curcumin complexed with phospholipids	clinical trials; fifty-two consecutive patients	activation of Nrf2 downregulates NF-κB controlled genes involved in inflammation, proliferation, survival, invasion, angiogenesis, and metastasis	[97]
**Sulforaphane**	1–100 μM	Panc1 and MiaPaCa-2 cell lines	activation of adenosine 5′-monophosphate-activated protein kinase (AMPK) by excessively generated ROS and subsequent increase in the Nrf2 nuclear translocation, which suppresses pancreatic cancer cell proliferation	[99]
	50 mg/kg, i.p.	transgenic mouse model	inhibition of tumor growth, consistent with the antiproliferative effects of SFN through ROS activated AMPK signaling pathway and NRF2 nuclear translocation	[99]
**Esculetin**	100 μM	Panc1, MiaPaCa-2, and AsPC-1 cell lines	directly binding to Keap1 and inhibition of its binding to Nrf2; reduction of ROS level, inhibition of cell growth, cell cycle arrest at G1 phase, and induction of apoptosis and loss of mitochondrial membrane potential	[100]
**Xanthohumol**	5 and 10 μM	Panc1 cell line	enhanced binding of Nrf2 to ARE sequence and increased protein level of Nrf2 correlated with decreased NF-κB expression; protein levels and inhibited proliferation	[71]
	5 and 10 μM	MiaPaCa-2 cell line	enhanced binding of Nrf2 to ARE sequence and increased protein level of Nrf2; in the combination with PEITC increased Caspase-3 and LC3 protein levels and inhibition of proliferation	[70]
**Resveratrol**	50 and 100 μM	Panc1 and MiaPaCa-2 cell lines	reduction in the level of NAF-1 and enhancement of the Nrf2 expression by inducing the accumulation of ROS, which contribute to cell death	[102]
	5 and 10 μM	Panc1 and MiaPaCa-2 cell lines	promotion of apoptosis via activation of Nrf2 and consequently downregulation of NF-κB	[70,71]
**Phenethyl isothiocyanate**	5 μmol/L	Panc-28, MiaPaCa-2, AsPC-1 cell lines	depletion of cellular GSH	[103]
	5 and 10 μM	MiaPaCa-2 cell line	activation of Nrf2 and its target genes by increased levels of p-JNK and decreased levels of p-GSK3β; increased Caspase-3 and LC3 protein levels	[70]
**Synthetic compounds**				
**Compound**	**Dosage**	**Model**	**Mechanism of modulation**	**Reference**
**Dexamethasone**	1 µM	Panc1 CSLC and PSN-1 CSLC (CSLC-cancer stem-like cells)	reduction of Nrf2 expression with significant decrease in GSH; increase in the growth-inhibitory effects of Gemcitabine and 5-fluorouracil	[104]
**Dimethyl Fumarate**	100 μM	MiaPaCa-2 cell line	a decrease in total Nrf2 and HO-1 corresponding with decreased DJ-1 protein levels	[107]
**PIK-75**	0.1–1 μM	MiaPaCa-2 and AsPC-1 cell lines; xenograft model	reduction of the Nrf2 protein levels and its transcriptional activity by proteasome-mediated degradation	[82]
**NSC84167**	1–10 μM	Panc1 and AsPC-1 cell lines; patient-derived pancreatic cancer cells and PDX tumor tissue	selectively targeting Nrf2-activated pancreatic cancer by inhibiting asparagine synthesis pathway; induction of apoptosis in Nrf2-activated pancreatic cancer cells independent of ROS	[60]

## 5. Conclusions

The role of oxidative stress and inflammation in pancreatic cancer development is well established. Due to the complex pathophysiology of pancreatic cancer, there is currently no effective treatment to counteract this damage. The dual role of Nrf2 in pancreatic cancer development, as well as its overexpression in pancreatic cancer, can be considered as a potential therapeutic target. The confirmation of this thesis has been found in the articles from the last decade.

It is now known that natural and/or synthetic compounds such as brusatol, curcumin, sulforaphane, and NSC84167 exert their effects by modulating the Nrf2 pathway. However, further elucidation of the detailed mechanism of molecular modifications by these compounds with respect to pancreatic cancer still needs to be found.

In summary, further research involving the use of inhibitors to block Nrf2 activity in malignant pancreatic tumors with constitutively active Nrf2 would be an important treatment strategy, and the use of human samples such as human biopsies or 3D cultures (organoids) can help develop new treatment strategies for this disease.

The authors hope to study the antioxidant and anticancer properties of various compounds that make them ideal candidates for treating these diseases, with the focus especially on their role as ROS scavengers, particularly their effect as Nrf2 signaling modulators.
